# The evolution of human-type consciousness – a by-product of mammalian innovation mechanism – a preliminary hypothesis

**DOI:** 10.3389/fpsyg.2025.1514077

**Published:** 2025-04-17

**Authors:** Uzi Ben Zvi

**Affiliations:** Independent Researcher, Nataf, Israel

**Keywords:** human consciousness, evolution, innovation, interface, neocortex, subcortical control

## Abstract

Human consciousness is often viewed as one of the pinnacles of evolution, with most theories positioning it as an upgrade of pre-existing cognitive skills. However, conscious perception, memory, action, and in some situations even decision-making, are often inferior—less complex, slower, and less accurate—than their nonconscious (subliminal) counterparts. The interface hypothesis challenges this perspective, proposing that human-type consciousness is not an advanced version of earlier cognitive capacities but a novel function that entered the arena of cognitive and operational processes and fundamentally changed its rules. According to this hypothesis, the neocortex emerged as part of an advanced innovation mechanism, where its unpredictable, chaotic activity is used to generate alternative patterns. The process of cropping these alternatives from the chaotic neocortex and mediating them to the constrained, goal-oriented, linear control system requires a serially functioning interface. Consciousness, it is suggested, arose as a byproduct or a side effect of this interface, eventually expanding its influence to a wide range of cognitive and operational functions. This perspective has significant implications for our understanding of human cognition, creativity, and the distinctive capacities of human consciousness, potentially bridging the gap between neuroscientific findings and phenomenological experiences of consciousness.

## 1 Introduction

For many years, modern Western thought and science almost completely avoided the question of consciousness. It was only toward the end of the last century that scientists began addressing this complex subject. Once awakened, scientific interest in consciousness gained significant momentum, leading to numerous attempts to reframe it within scientific knowledge. However, as Thomas Metzinger has recently observed, “Three decades after the Association for the Scientific Study of Consciousness was founded in 1994, we still do not even know (or cannot agree on) what precisely it is that needs to be explained” (Metzinger, 2024).

The interface hypothesis, proposed in this article, offers a novel perspective on the evolution and function of consciousness. It suggests that consciousness emerged as a byproduct of an interface between the brain’s innovation system and its automatic control system. This hypothesis does not stem from, nor directly align with existing theories. Therefore, I will first present this proposal and then compare it with some of the leading theories.

Since there is no gold standard for the term “consciousness,” human-type consciousness in this exposition will apply to the situation or process in which humans can:

1. Know they receive sensory impressions, and distinguish between them.
2. Know they perform physical actions, and identify each action.
3. Know they feel emotions, and distinguish between them.
4. Know they want, think, and even know.
  And as a consequence of all these:
5. Voluntarily and consciously influence the extent, timing, and manner in which these functions are manifested.

To distinguish these capacities from other phenomena that are often called “consciousness,” I refer to it as “knowing consciousness.” Throughout this essay, the term “consciousness” will refer exclusively to this knowing consciousness.

In ancient Buddhist tradition, consciousness is counted as the sixth sense, which, unlike the five outwardly directed senses, monitors internal events (The Buddhists seemed to ignore the nonconscious capacities of proprioception and interoception, which also monitor internal bodily events). Unlike the five senses, consciousness does not necessarily correspond to external events, or even to internal somatic ones. When it does, it does not relate directly to these events but to their neuronal representations generated in the brain during data processing from the other senses. The ability to monitor such neuronal events extends to non-sensory neuronal events, such as thoughts and emotions, which may occur independently from ongoing external or somatic occurrences.

However, the act of knowing itself—the monitoring of such occurrences—is also a neuronal process and, as such, can also be monitored. That is, one can know that they know. It is also worth noting that, at least in humans, the act of knowing is intimately related to selfhood. The ability to observe inner neuronal occurrences is inseparable from the experience of internal separation between subject and object. The entity that knows—the “I” or the “self”—is the subject, while all other parts and functions—body, actions, emotions, will, and thoughts—are conceived as “mine,” that is, as objects.

Knowing is indeed the basic function of consciousness, but it is not the only one, and probably not the most significant. The main impact of consciousness (and knowing) on daily life comes from its active contribution to the controle of daily activities, sophisticated learning, and the evolution of higher cognitive skills such as thought, language, and higher emotional functions.

### 1.1 The paradox of consciousness’ serial functioning

Knowing consciousness appeared at a relatively late stage of evolution and, in this respect, can be regarded as one of its summits. However, this assertion is challenged by some of its basic characteristics. In terms of its effect on one’s capacities—such as perception and action—consciousness often appears detrimental rather than beneficial. Conscious involvement may severely impair the speed and scope of performance it is “rather slow, prone to error, unable to perform simultaneous tasks, and quite limited” (Baars, 1993) compared to the nonconscious control system. Unlike consciousness, the more “primitive” control system is capable of simultaneously receiving a large number of different stimuli, weighing them, and generating responses whose speed and eloquence conceal their complexity.

The word “conceal” highlights the fact that consciousness is unable to follow this complexity—neither in sensory input nor in the operative output of the nonconscious control system. Shaped and perfected over hundreds of millions of years of evolution, the nonconscious system operates in a parallel, multichannel mode, whereas consciousness operates in a far less sophisticated, single-channel, serial mode (Rabinovich et al., 2020). Consciousness’ operation is inferior in many respects. At any given moment, consciousness can perceive only one, or at most a small number, of messages, and that at a relatively slow pace. Therefore, when it tries to follow the rapid and complex operations of the control system, it perceives only their general outlines or final outcomes. Accordingly, in its operative aspect, consciousness cannot simultaneously control several gestures and integrate them into one coordinated action.

From an evolutionary point of view, the late emergence of the serial non-efficient and energy consuming operation mode of conscious control (Holroyd, 2024), raises a disturbing question: evolution generally progresses from the primitive, which is simpler and less efficient, to the more advanced, which is expected to be more complex, sophisticated, and efficient. In the case of consciousness, it looks as if evolution have regressed. While seriality is the building block of any goal-oriented actions, almost every such action, requires a simultaneous application of several serial channels, hence parallel operation. The single-channel, serial mode of operation typical to consciousness (Marois and Ivanoff, 2005) appeared hundreds of millions of years after the nervous systems of vertebrates had already mastered the capacity for parallel control of muscle operation and parallel processing of sensory input. This evolutionary question is compounded by the difficulty of locating this serial mode of operation in the brain’s anatomy. We know of brain parts, such as the cerebellum, yielding serial-parallel operation, and others, mainly the neocortex, that operate in a reentry mode, yielding chaotic results, but we cannot point to a location that function in a single-channel, serial way, yielding the characteristic behavior of consciousness. The seriality we do find is usually a part of a wider parallel operation, and unrelated to consciousness.

The incompetence of consciousness in operative control corresponds with the increasingly established understanding that it is difficult to identify an operative role in daily human activity that necessitates consciousness (Hassin, 2013). All the basic functions of developed animals, from metabolic functions to sensory and motor activities, emotion, and decision-making, evolved long before the appearance of human consciousness. These functions work well in organisms that do not need to know what they are doing, and in humans, most of these functions can be carried out without consciousness being involved (Ginsburg and Jablonka, 2019). This is not surprising when it comes to motor or sensory functions, but it turns out that even high-level human functions, such as complex decision making (Dijksterhuis and Strick, 2016), chess-playing (Dreyfus and Dreyfus, 2005), or arithmetic (Hassin, 2013), which presumably could not be acquired without consciousness, can eventually be carried out without it—and in the cases of savants (Heavey, 2013) or bullet chess players, more quickly and efficiently. This does not mean that consciousness is not involved in such functions, but that in many cases its contribution is marginal, unnecessary, or even detrimental.

Consciousness is an inseparable part of human existence and the exclusive tool we use for self-study and exploration. This may explain why we tend to take its very existence for granted and ignore its disturbing characteristics. The evolutionary path for the emergence of consciousness proposed here may explain its main peculiarities and provide answers to some of the questions they raise.

### 1.2 This argument is divided into six parts

1. Present the inevitable counteraction between the organism’s basic (subcortical) control system, which must provide an accurate deterministic response to current challenges, and its innovation-producing system, which must find new ways to meet new challenges.
2. Argue that the evolution of the neocortex and its typical chaotic activity was driven by the need to enhance innovation without disrupting the linear-parallel dynamics of the subcortical control system.
3. Suggest that the interface between these two modes of operation consists of simple serial messages and directions drawn from the chaotic innovation generator and introduced into the control system.
4. Point out the similarity between the anatomical and functional characteristics of this interface and those ascribed to human consciousness, arguing that this similarity suggests consciousness is a byproduct of the interface.
5. Compare the interface hypothesis with prominent existing theories.
6. Explore some of the implications, limitations, and open questions raised by this hypothesis.

## 2 Neural Architecture of Innovation and Control Systems as the Foundation of Consciousness

### 2.1 Stabilization vs. exploration in the mammalian brain

#### 2.1.1 The evolution of innovation capacity

Ever since the beginning of life on earth, the immediate task of any organism has been to preserve itself (Bich et al., 2016), at least long enough to create offspring. In parallel, every species faced an ever-growing need to adapt to changing conditions, hence, to alter the constraints that preserved it. For over three billion years, both the stabilization mechanisms (which preserve the individual organism) and the variation-generating processes (Payne and Wagner, 2019: Kumawat et al., 2025) (which promote the evolution of the species) evolved within the domain of cell biochemistry, particularly in the genes (LeDoux, 2023). This is also where the mechanisms that maintain the equilibrium between these two opposing trends have evolved and been perfected. Genes remain the most important arena of evolutionary processes (Jablonka and Lamb, 2014). However, in parallel, animals with a complex central nervous system have developed a similar dynamic within the nervous system of the individual (Uddin, 2021). In nervous systems, as in genetic systems, two opposing trends function side by side: the first aims to strengthen and stabilize already-established patterns that can cope with a variety of more or less predictable challenges, and the second provides flexibility and creativity necessary for finding solutions to new challenges. Like the dynamics of genetic variation, this flexibility is enabled largely by random “noise”—neural activity that does not serve a concrete specific purpose and is not bound to the organism’s pre-established modes of action. From this noise, new response variations may emerge (Szilágyi et al., 2017), one of which may prove useful and become a new default for similar challenges.

#### 2.1.2 The equilibrium between conservation and innovation: a power struggle

Both the ability to stabilize and maintain optimal functioning (exploitation, stabilization) (Marder and Goaillard, 2006; Cohen et al., 2007) and the use of random variations to search for alternative modes of action (exploration) are basic traits of all brains, and consequently can be found in all levels of the human brain. Guarding the equilibrium between these trends has always been complex, but in more sophisticated brains, the challenge is greater by far. The brain is a noisy system at any level of examination, primarily because its main building block—the neuron—is inherently noisy (Faisal et al., 2008). The ability to predict the output of an individual neuron in response to a specific input is never absolute. The noise (or stochasticity, variability, or fluctuations) in the functioning of a single neuron, amplified by the brain’s enormous complexity, could be catastrophic since even the slightest inaccuracy may divert the whole system from its intended route. Such dynamics certainly provide fertile ground for innovation, but the brain is first and foremost the organism’s control system, which must maintain a high level of reliability. Despite this noise, the brain carries out a variety of incredibly complex activities requiring precisely coordinated deterministic output, both in volume and timing.

It is plausible to posit that when deterministic reliability and exploratory activity operate together in time and space, they oppose each other, as each one must neutralize the influence of the other to manifest itself in full. As both trends evolve and grow stronger, the organism may need more and more energy to maintain equilibrium between them. It is like an arm-wrestling competition in which the stronger the competitors, the more energy is spent, but as long as equilibrium is maintained, the result remains the same—neither prevails. In mammals, whose survival strategy relies on large brains and high flexibility, this mutual neutralization may lead to an intolerable waste of energy, requiring another solution.

We can examine this challenge, and the solution that nature found, by comparing it to human organizations facing similar challenges. Much like biological systems, modern industry must constantly stabilize and optimize its production processes while simultaneously renewing itself by exploring alternative products and methods to remain competitive (Benner and Tushman, 2003). Many organizations resolve this contradiction by creating an almost complete separation between these functions. Production lines are designed to minimize noise, channeling erratic forces into a functioning machine that produces a well-defined product as efficiently as possible. At the same time, these organizations run dedicated R&D units tasked with finding solutions to unforeseen needs, predicting future demands, and preparing new modes of action. These R&D units are less constrained, as they are not expected to deliver immediate pre-determined results. Since their activity is separated from the production line, they do not disrupt it, and there is no need to restrict them.

The model I propose suggests that the subcortical parts of the brain, primarily the basal ganglia and the cerebellum, control the “production line.” They take most of the responsibility for ongoing interactions with the environment, while the newer part that evolved in mammals, the neocortex, primarily serves the growing need for innovation (Dietrich, 2004).

### 2.2 The division of roles between the neocortex and the cerebellum

The neocortex and cerebellum together occupy about 90% of total brain volume and contain about 80% of the brain’s neurons, though their distribution between these two organs is uneven (Herculano-Houzel, 2010). The neocortex occupies roughly four-fifths of the brain’s volume but contains only about one-fifth of its neurons, while the cerebellum, which occupies about one-eighth of the brain’s volume, contains two-thirds of its neurons. Based on either volume or the number of neurons, it is reasonable to conclude that most brain functions are shared between these two structures, although the division of roles between them is less obvious.

In most bodily systems, it is possible to see a clear correlation between the anatomy and physiology of an organ and the functions it performs. However, in the case of the brain, this correlation is often vague, and in many cases, it does not lead to clear conclusions. With this in mind, I will nevertheless attempt to compare the different anatomical structures of the neocortex and cerebellum to what we know about their modes of operation, with the aim of drawing conclusions about their respective roles.

#### 2.2.1 Evolutionary perspective: the ancient cerebellum

The cerebellum is evolutionarily ancient, appearing in all vertebrates, beginning with fish, amphibians, and reptiles, in which it (along with the basal ganglia) constitutes the highest control level for the animal’s behavior. Compared to other parts of the brain, especially the neocortex, the cerebellum’s microanatomy is marked by a distinct spatial organization. This three-dimensional crisscross array maintains a clear directionality and regular patterns of connectivity at each dimension (Apps and Garwicz, 2005). This structure is similar to the internal organization of dedicated organs like, for example, the kidney, or to a well-designed machine or production line. This clear directionality likely serves the vital function of overcoming neuronal “noise” (i.e., the unpredictable activity of single neurons) by channeling it into a tightly controlled set of commands. This directionality corresponds with the cerebellum’s typical activity pattern, known as “feed-forward” processing (Bastian, 2006), in which input moves mainly in one direction, with minimal reentry and recurrent processing, and limited interaction between parallel streams.

The cerebellum primarily serves as the brain’s executive part, controlling mainly complex motor tasks but also cognitive emotional and autonomous (Schmahmann, 2019) functions. Almost every physical gesture, even seemingly simple ones, involves tightly timed and measured activation of many muscles in parallel sequences that influence each other and advance together toward a predefined goal. For this purpose, the cerebellum must quickly and accurately process and weigh a large number of signals, mainly proprioceptive and visual, to produce a precise and well-timed sequence of operating instructions for the muscles. The anatomical structure and neural dynamics described above seem well-suited to producing the kind of serial-parallel output, enabling simultaneous management of numerous serial actions that must be coordinated and integrated into a single goal-directed action.

#### 2.2.2 The neocortex: a newcomer in evolution

When it comes to the neocortex, linking its microanatomy to the purposes and functions it serves is much more difficult. The neocortex is a brain structure unique to mammals, making it a relatively recent evolutionary phenomenon. When the neocortex first appeared, it was smaller and less sophisticated than brain structures that preceded it by hundreds of millions of years (Kaas, 2011). By then, basic cognitive functions like motor control, sensory perception, basic emotions, and decision-making were already performed by older brain structures. These structures, particularly the cerebellum, have been preserved in mammals. The further evolution of some of these structures (such as the tectum) has slowed or even receded, likely due to the neocortex taking over some of their functions (Striedter, 2005). However, the cerebellum continued to grow in size and probably also in sophistication, alongside the growth of the neocortex (Barton and Venditti, 2014) and most its functions appear to have been retained. This suggests that the exceptional evolutionary expansion of the neocortex did not come at the expense of the older control system, but rather in addition to it.

Against this background, one must ask what needs drove the evolution of the neocortex. In humans, the neocortex seems to play a crucial role in almost every aspect of behavior. For example, a lesion in the human motor cortex inevitably leads to severe and usually irreversible motor damage. However, in other primates, the effect of similar lesion can be temporary and recoverable, while in non-primate mammals, such a lesion has little effect (Lopes et al., 2023). Cats, dogs, and mice that have had their entire motor cortex removed retain their motor abilities almost unimpaired. Similarly, the role of the neocortex in sensory processing varies between primates and other mammals. In primates, including humans, damage to the primary visual cortex (area V1) causes cortical blindness (blind sight), but in other mammals, its effect is barely noticeable (Tehovnik et al., 2021). From these differences, we can conclude that the neocortex’s vital role in assuring ongoing control of movement and vision is a relatively recent phenomenon. Even if most mammals show evidence of some neocortical involvement in perception and control processes, we can conclude that the neocortex originally evolved to meet other needs.

Hints about the neocortex’s evolutionary role can be drawn from the anatomical and functional differences between the neocortex and the cerebellum. Compared to the highly organized and directionally strict anatomy of the cerebellum, the neocortex, which is by no means less complex, shows much less organization hence, greater degrees of freedom. Although the neocortex shows some directionality, it is only in the vertical dimension and even there it is not quite robust. Most neocortical neurons are large, branched, and complex, with far less constrained and directed connectivity. This type of anatomical structure does not seem well-suited to producing precise, goal-directed, deterministic output—i.e., for control purposes. Instead, the non-constrained, noisy, chaotic dynamics that arise from the non-deterministic activity of individual neurons are enhanced by the neocortex’s overall complexity. Therefore, the neocortex anatomy seems more appropriate for producing the random variations necessary for innovation.

Chaos, one of the main characteristics of the neocortex’s dynamics, manifests in terms such as self-organization, nonlinearity, sensitive dependence on initial conditions, criticality, stochastic fluctuations (Singer, 2015; Tozzi et al., 2016), spontaneous activity, and reentry dynamics (Edelman and Gally, 2013). Together, these characteristics explain why neocortical activity persists even in the absence of external input and why its output cannot be predicted from the input it receives. This mode of action, which resembles background noise, seems useless for goal-oriented control purposes, but its potential advantage may lie in generating new, alternative modes of action. As far as we know, the neocortex plays a primary role in simulating situations, past, and future, and even imagined ones (Buckner and Carroll, 2007). This ability contributes to the invention of new variations on old patterns of action or new responses to novel challenges.

Indeed, a significant portion of neocortical activity is dedicated to the default mode network (Raichle et al., 2001; Menon, 2023). It operates almost non-stop and, generally speaking, its intensity is inversely proportional to the performance of concrete tasks. Although the default network is usually hardly affected by ongoing activity, its activity subsides when the brain’s resources must be mobilized for a particularly demanding challenge. The only situation in which the default network’s activity positively correlates with external challenges is during creative problem-solving, where peaks of activity have been observed before solutions emerge (Broday-Dvir and Malach, 2021). Conversely, interruptions to the default network reduce creative problem-solving ability (Shofty et al., 2022; Bartoli et al., 2024).

This behavior of the default network leads to the assumption that its noisy activity, which lacks an immediate operational role, is not an excess or a side effect (“bug”) but rather the primary reason for the neocortex’s evolution (“feature”). The neocortex can be described as a kind of variations generator (Szilágyi et al., 2017), perfected in mammalians over hundreds of millions of years to provide alternative solutions to challenges without ready-made answers. This variations generator can be regarded as the biological equivalent of an R&D department, separated from the production line, equivalent to the subcortical control system. Hence I suggest that the separation of these two parts of the brain allows for the chaotic, energetic activity of the neocortex to proceed without disrupting the control system’s vital functioning.

However, the alternative modes of action generated by this chaotic activity are useless unless they are introduced and assimilated into the control system. The question, therefore, arises: how are the two connected? What kind of interface delivers the products of this highly energetic, noisy activity into the constrained control system?

Returning to the R&D metaphor: in order to integrate their innovations into the production line, savvy R&D people have to organize the new proposal in a simple and comprehensible manner, breaking it down into stages. They then have to arrange a dedicated event during which the current production activity is halted and conduct a step-by-step presentation of the new process to the production team. Once the new process is introduced, the production team, more skilled in their craft, takes over to test the proposal and provide the final polish.

Is it possible to draw similarities between this description and the interface between the innovation production processes in the neocortex and the subcortical control system? Probably yes, and as I will demonstrate hereby, quite a few of them.

### 2.3 The Interface

#### 2.3.1 Seriality as a common language

The neocortex, in its role as a variation generator, operates in a highly energetic, chaotic manner. Therefore, a direct assimilation of its output into the constrained subcortical control system, which operates in a linear, parallel, and energy-saving manner, would likely disrupt the latter’s functioning. In my view, the serial mode of action characteristic of consciousness evolved as a solution to this problem.

As noted, almost all brain activity is shared between the “wild” chaotic activity of the neocortex and the constrained, sophisticated parallel activity of the cerebellum, necessary for simultaneous control of multiple series of commands. However, the story that our conscious subjective experience tells us does not fit either of these modes of action. Conscious controlled motor functioning, as well as the way we think and experience ourselves follow the same single-channeled serial mode that we apply when writing or reading these sentences. This conscious human version of serial activity is undoubtedly a new evolutionary phenomenon, and as such, we tend to consider it one of the peaks of evolution. However, the late appearance of such single-channeled seriality, which is less complex, efficient and sophisticated than the parallel activity that preceded it, suggests a case of regressive evolution. Such an evolutionary event should have occurred for a good reason It was acknowledged by Baars (1993), but seems to be ovelooked or unnoticed by scientists and philosophers studying consciousness. The explanation I propose is that the evolutionary drive that brought about this serial activity is its essential role in the interface between the variation generator and the control system.

Mediating between random chaotic activity and linear, goal-oriented activity is by no means trivial. In simple living systems and in small human organizations it may be possible without a dedicated interface, but more complex systems may require one. An additional process is needed, analogous to the preparation of operating instructions in the R&D department for presentation to the production floor team. It is likely that transferring information from the chaotic system to the parallel control system requires passing through the common building blocks of these two modes of operation—simple, linear, serial messages. The multidimensional and noisy chaotic message produced in the neocortex must be reduced, flattened, quieted, and stabilized, so that only its basic outlines remain. The relevant information sampled from the chaotic system becomes a simpler message, similar in nature to the basic components of parallel activity. This similarity enables its use as a building block, or skeleton, for the construction of a new parallel operation pattern in the control system.

#### 2.3.2 The anatomy of the Interface

If the interface I propose exists, we know very little about it. In principle, its existence does not require a specific anatomical location. It could, like many other functions, be executed by network activity. However, if we insist on looking for an anatomical location associated with this interface, the leading candidate is probably **the claustrum**.

The claustrum is a brain structure described in recent literature as “intriguing” (Atlan et al., 2018) and “enigmatic” (Torgerson and Van Horn, 2014), and its functional role “remains unknown” (Atilgan et al., 2022) and “least understood” (Nikolenko et al., 2021). The two claustra (one in each hemisphere) are thin, sheet-like structures that partition the inner part of the neocortex, the insula, and the basal ganglia. The claustrum is characterized by extensive connectivity; relative to its tiny volume, it is the brain structure most connected to the rest of the brain. It is linked to most parts of the neocortex, particularly those making up the default network, which, as I suggest here, is also home to the variation generator. Despite its extensive connectivity, the claustrum itself is less complex than most other brain structures, consisting mainly of one type of nerve cells. Although it is risky to draw conclusions about its activity, it is tempting to suggest a relationship between its structural simplicity and the serial activity of the interface, which is also far less complex than other parts of the brain. Crick and Koch (2005), who were the first to associate the claustrum with consciousness, proposed that the neocortex is entirely represented in the claustrum, but likely in a partial and “diluted” manner, consistent with the hypothesis that the interface “flattens” the multidimensional messages from the variation generator.

#### 2.3.3 The operation pattern of the interface

Even if the claustrum is, indeed, the anatomical location that hosts the interface, our ideas about its modes of action remain largely speculative. To concretize the discussion, I will focus on the interface’s basic and primary function—motor innovation.

Muscle control is the main function for which the central nervous system originally developed, and even in the most advanced brains, this is still its primary function. All other operative functions (as distinguished from sensory ones) evolved much later, presumably based on the motor control circuits already in place (Wolpert et al., 2011). For this reason, and because motor activity is easier to study, understanding the mechanisms of motor innovation may be key to understanding innovation in other domains.

A closer look at how new motor patterns are adopted reveals that learning and assimilating new possibilities is not achieved through transmission of operating instructions, but through a demonstration of the action itself (Hodges and Williams, 2012). This can be seen in how a person learns the physical gestures necessary for using an unfamiliar tool. Usually, a verbal explanation, no matter how detailed, will not yield the desired result. Demonstration by someone who already knows the action will likely yield better results (probably with the help of mirror neurons). When these two methods are insufficient, the instructor can use direct physical guidance—holding the trainee’s hands and leading them through the required movement. An even clearer example is how one learns new patterns of action in disciplines such as the Alexander Technique or the Feldenkrais Method (Feldenkrais, 1981). In both cases, the information is transferred by the teacher without the student consciously knowing what or how they learned. When the body moves in a new and unfamiliar way, learning occurs in the nonconscious control system, which assimilates and stores the new action pattern for later use. The student is only aware of the end result—the bodily sensation of the action—so the reported feeling is often that “the body learned”.

The primitive, nonconscious control system is the default tool for controlling skeletal muscles, and it does so with speed and efficiency beyond the resolution of conscious perception. Therefore, I suggest that for the interface to interfere in ongoing control and demonstrate a new bodily function, it must overcome this control. Minimally, it must inhibit the output of the upper level of the control system; otherwise, the control system will take over and perform the operation in its familiar way. Only when this level is muted can the interface take its place and, in its slow and clumsy way, direct the action of the body. However, the interface’s limited control capacity is insufficient to simultaneously regulate and time every single muscle. In fact, this is also likely true for the upper control level it replaces. It does not pass down detailed commands, only general ones. These commands likely refer to “packages” of actions, while the details are coordinated by lower levels, probably located in the brainstem or spinal cord, as can be deduced from experiments with decorticated animals (Perret, 1976).

The hypothesis I present here assumes that only the output of the upper control level is inhibited, not the input it receives. When the muscles are activated under the control of the interface and perform a body gesture, the proprioceptive feedback produced by the muscles and joints continues to flow upward. It can reach the highest nonconscious control level, which is otherwise inactivated but can still monitor and identify the new pattern. In this way, the control system can learn to imitate the sequence of movements, and once the pattern becomes familiar, it can improve and optimize it into a skillful act without further intervention from the interface.

This proposed description of the interface’s functioning refers only to its output—the transmission and assimilation of changes. It does not relate to its input, i.e., how the interface scans chaotic activity and “crops” the promising variations. The primary reason for neglecting the input side is that we have no direct experience of what happens there. All we know is that there is a circumstantial relationship between increased activity in the neocortex and the emergence of creative solutions (Broday-Dvir and Malach, 2021). The description of the output side is also only partially based on empirical facts and relies mainly on functional logic and inference that (to me) seem reasonable.

### 2.4 The Interface and consciousness – a wide range of similarities

Up to this point, I have described the proposed dynamics of the interface between the variation generator and the automatic control system. However, as I will demonstrate below, the interface and knowing consciousness are closely related, to the point that in the above description, the word *interface* can often be replaced by the word *consciousness*.

It is important to note that when we examine the relationship between consciousness and brain activity, it is not easy to distinguish between the variation generator and the interface described here. The entire cortical activity, as a variation generator, is hidden from our conscious experience, and what we can witness is only its outcome after it is transmitted to the interface and appears seemingly “out of nowhere.” The close temporal proximity of these two occurrences is misleading. This is why it is generally agreed that the chaotic activity of the neocortex is associated with consciousness, even though by nature, consciousness is a linear and serial phenomenon.

#### 2.4.1 The similarities are as follows

With this in mind, I will present several points of similarity between what we identify as consciousness and the production and assimilation of alternative patterns of action:

- The location in the brain: There is no consensus on a specific part of the brain where consciousness resides, but it is generally assumed to be related to the thalamo-cortical system (Llinás et al., 1998), especially the frontal lobe, and/or the claustrum (Crick and Koch, 2005). It is commonly assumed that the thalamo-cortical system is also the anatomical and functional source of innovation (Beaty et al., 2014; Broday-Dvir and Malach, 2021), and, according to my hypothesis, the same is true of the claustrum.
- Relation to the default mode network: My hypothesis proposes that innovation is the survival benefit that promoted the evolution of the neocortex, and in particular the default mode network and its chaotic activity. There is also scholarly consensus that this network is involved in consciousness (Raichle, 2015). The network is characterized, among other things, by almost nonstop activity, with no visible relation to any particular operational need or received external messages. The similarity of this activity to the incessant stream of thoughts—one of the main manifestations of consciousness—is hard to ignore. Like the activity of the default mode network, thoughts swirl continuously, often without external stimulation or apparent direct benefit.
- Separation between consciousness and the control system: One of the main characteristics of knowing consciousness is its operation in a “virtual space,” independent of current sensory input and operative activity. For example, the automatic control system can engage in complex activities such as driving, walking, or even playing piano, while consciousness, mainly in the form of thoughts, may be disconnected from it and occupied with other matters. According to my hypothesis, this kind of separation is a key characteristic and essential prerequisite of the innovation system, which must operate separately and in parallel with the control system.
- Serial mode of operation: Our conscious experience, both of the world and of ourselves, is less rich and sophisticated than the nonconscious tools we use to monitor the world and carry out routine behaviors. Against the background of the brain’s sophisticated, complex, parallel, and multichannel operation, the late appearance of the serial mode of operation in consciousness is a nontrivial phenomenon that requires explanation. By now, the only explanation is the role it plays in the interface between the variation generator and the control system.
- Dynamics of reentry: According to some theories of consciousness (Seth and Bayne, 2022, Edelman, 1989, Edelman, 1993), the dynamics of reentry—an essential component of the variation generator—also form the basis of consciousness. I should again point out here the apparent contradiction between this statement, which links consciousness to chaotic activity, and the description in the previous section, which links consciousness to the serial mode of operation. This contradiction can be explained by the fact that consciousness is serial in nature, but the interface hypothesis presents it as a necessary step for assimilating the products of nonconscious chaotic activity. As a result, every instance of consciousness, which is serial in nature, is only the “tip of the iceberg”—a partial and limited representation of a previous, non-conscious chaotic cortical activity that led to it.
- Inhibition of the automatic control system: When one draws attention (that is, conscious awareness) toward a certain sensory or motor activity, the upper level of the control system is almost necessarily disabled, and consciousness takes its place. In the case of a familiar and common activity, such as blind typing or even jumping from rock to rock while crossing a stream, the intervention of slow and cumbersome consciousness hinders the automatic system, and the quality of performance deteriorates. The only context in which this dynamic plays a positive role is in innovation, as part of the interface’s modus operandi. When one must learn a new pattern not yet assimilated by the automatic system, the intervention of consciousness becomes useful, and often even essential.
- Functioning in an emergency: In contrast to the situations addressed above, when one faces a demanding or critical challenge, the automatic control system can free itself from the inhibiting influence of consciousness and function more quickly and efficiently. This behavioral phenomenon corresponds with the phenomenon I mentioned in the context of brain activity: when intensive, goal-oriented action is required, the default network’s activity (which, as I propose, is the main source of innovation) is inhibited and silenced.

#### 2.4.2 Therefore

Folk wisdom says that if it looks like a duck, walks like a duck, and quacks like a duck, it is probably a duck. Alone, none of these similarities is sufficient to rigorously establish a link between consciousness and the interface. But taken together, they make the connection quite plausible. Since the mammalian innovation system far preceded human consciousness, I propose the following hypothesis: **originally, knowing consciousness appeared as an aspect or byproduct of the interface between the innovation system and the automatic control system**.

However, it is important to emphasize that this statement does not point to an identity between the interface and consciousness. All mammals possess a neocortex, most have a claustrum (excluding monotremes), and many have a significant default mode network. Given these shared neural structures, it’s plausible to posit that many mammals possess similar innovation systems with similar interfaces. However, almost none of them exhibit signs of human-like consciousness. I hypothesize that a certain threshold of complexity and intensity must have been crossed for the emergence of consciousness, but it’s currently impossible to determine which, if any, other mammals have reached this threshold.

#### 2.4.3 Interim summary

The interface hypothesis of consciousness proposes that knowing consciousness emerged out of one of the stages of the process of assimilating innovation—specifically, the one that enables the communication between the variation generator and the control system. Therefore, its appearance is inseparable from the chaotic activity of the neocortex. However, we can directly experience only this last stage, likely because consciousness is an inseparable part of it. In other words, consciousness is related to the chaotic activity of the neocortex because it is tightly connected to the interface that transforms and introduces the outcomes of this activity into the control system. The chaotic activity itself, as well as most parts of the control system, remain inaccessible to consciousness, so what we perceive and report is only this intermediate stage of a larger process. Thus, while consciousness is closely related to and influenced by neocortical processes, it is not a direct result of them.

### 2.5 The Interface hypothesis and other theories of consciousness

In the absence of a commonly accepted definition of consciousness, attempts to describe, explain, and relate it to adjacent domains of knowledge almost inevitably fall into a common methodological trap: the tendency to reverse the process by not only explaining consciousness but also revising its definition to fit other phenomena presumed to be associated with it. This tendency aligns with Anil Seth and Tim Bayne’s statement (Seth and Bayne, 2022) that “One of the main reasons why ToCs (Theory of Consciousness—U.B.) ‘talk’ past each other is that they often have different explanatory targets, for they focus on different aspects of consciousness”.

Scientific efforts to describe, define, and explain consciousness generally proceed in three main directions, which are sometimes combined:

1. The phenomenological approach aims to describe consciousness, distinguish it from other cognitive traits, and ultimately define it. It is generally a qualitative description of observable behavior, often obtained through self-observation. In principle, it is a necessary starting point for the other two.
2. The neuroscientific approach, grounded primarily in data obtained through various imaging techniques. Seeks measurable neural phenomena that may correlate with consciousness (as previously described or defined) or explain it.
3. The evolutionary approach speculates on possible evolutionary paths that could have led to the emergence of consciousness. It often incorporates aspects of the other two approaches and sheds new light on them.

#### 2.5.1 Phenomenological approach

The phenomenological approach has been adopted by numerous researchers, each offering distinct perspectives on consciousness. These theories can be broadly categorized as follows:

Higher-order theories: Initially proposed by Rosenthal (1993) and later reviewed by Genaro (2004), these theories suggest that a mental state becomes conscious when a supplementary level of processing occurs, such as perception or thought.

Multiple processes theories: Dennett (1991) views consciousness as the outcome of interactions between different processes occurring simultaneously in different parts of the brain.

Predictive processing theories: Graziano (2019) emphasizes the importance of the brain’s ability to relate to imagined futures. Hohwy and Seth (2020) sees the capacity to predict the body’s internal states as the source of consciousness.

Social cognition theories: Humphrey (1992) argues that the origin of consciousness lies in the capacity to create a theory of mind for others, enabling complex social interactions.

Regulatory function theories: Solms (2021) views consciousness as a regulatory function, primarily for emotions and drives, allowing organisms to more effectively manage their needs and desires.

The interface hypothesis shares commonalities with higher-order theories but differs in its perspective on the role of consciousness. Unlike many other theories, it does not view consciousness primarily as a tool for regulation and control. Furthermore, the interface hypothesis uniquely addresses the question of the limited serial functioning of consciousness, a feature often overlooked by other phenomenological theories.

#### 2.5.2 Neuroscience-based theories

The two leading neuroscientific theories of consciousness are Global Workspace Theory (GWT) and Information Integration Theory (IIT).

Global Workspace Theory (GWT), originally proposed by Baars (1988) and later expanded by Dehaene (2014), suggests that consciousness arises when information is widely broadcast across the brain, allowing different areas to integrate and share this information. In this theory, consciousness functions as a “global workspace,” where integrated information becomes available for decision-making, memory, and action.

Information Integration Theory (IIT), developed by Tononi (2008) and refined by Oizumi et al. (2014), posits that consciousness corresponds to the amount of integrated information within a neural system. According to IIT, the degree of interconnectedness and complexity in a brain’s network determines its level of consciousness.

Other notable neuroscientific approaches include:

Recurrent processing theory: Proposed by Lamme (2006, 2010, 2018), this theory posits that consciousness arises when sensory information is subjected to recurrent processing rather than just feedforward processing.

Reentry theory: Proposed by Edelman (1989, 1993), this theory suggests that consciousness emerges from the dynamic integration of information across multiple brain regions through reentrant interactions.

All these theories provide valuable insights and are deeply rooted in neurological data, but they face some significant limitations:

- Lack of clear demarcation: These neurologically-oriented theories lack a robust phenomenological foundation, resulting in an unclear distinction between conscious and nonconscious functions.
- Focus limited to cortical functioning: These theories regard the neocortex and its functions as the sole seat of consciousness, overlooking the discrepancy between the chaotic functioning of the neocortex and the serial functioning of consciousness.
- Overlooking the seriality paradox: With the exception of Baars (1993), these theories do not address the paradox of consciousness’s serial processing mode. However, while these neuroscientific theories provide essential groundwork, they neither conflict with nor directly support the interface hypothesis.

#### 2.5.3 Evolutionary theories

Evolutionary theories of consciousness attempt to explain how, why, and from which preceding traits consciousness emerged. If we ever reach a broadly accepted evolutionary theory, it may contribute to a better understanding of the essence of consciousness and the actual role it plays. By now, the different propositions do not seem to converge into a unified theory.

Here are several prominent theories, that can be categorized based on their primary focus:

Emotions: Damasio (2010) and LeDoux and Brown (2017) emphasize the importance of emotions and bodily states in the evolution of consciousness. LeDoux focuses on how consciousness integrates survival-related behaviors, while Damasio highlights the role of internal bodily representations and homeostasis in generating the subjective feeling of consciousness.

Social cognition: Dunbar (2016) and Donald (2001) emphasize the role of social relations in the evolution of human cognition and consciousness. Dunbar attributes the emergence of consciousness to the complexity of social relationships, while Donald argues that higher consciousness, including episodic memory and symbolic thinking, evolved to support social interactions and cultural transmission.

Learning: Ginsburg and Jablonka (2019) focus on Unlimited Associative Learning as a “minimal” consciousness and a precursor to more advanced one.

Attention and self-representation: Graziano (2019) suggests that consciousness evolved as an internal model created by the brain to represent its own attention processes.

The interface hypothesis differs fundamentally from these evolutionary theories in several ways:

1. It proposes that consciousness emerged as a byproduct, rather than as a direct continuation of preceding cognitive traits or a response to survival needs.
2. It questions the direct contribution of consciousness to survival, which is often presumed by other evolutionary theories.
3. It offers a unique explanation for the serial nature of conscious experience, framing it as a necessary feature for translating chaotic, parallel neural activity into linear, implementable actions.

#### 2.5.4 Conclusion

While existing theories offer valuable insights into various aspects of consciousness, the interface hypothesis provides a novel perspective on its evolutionary origin and functional role. By proposing that consciousness stems from the interface between innovation and control systems, the hypothesis offers a framework that could potentially integrate aspects of phenomenological, neuroscientific, and evolutionary approaches. Future research may help clarify the relationships between these theoretical approaches and lead to a more comprehensive understanding of consciousness.

## 3 Implications, limitations, and open questions in Interface theory

The emergence of human consciousness as a byproduct of another evolutionary process is by no means an exceptional evolutionary event. As a rule, new anatomical or functional features evolve through tinkering (Jacob, 1977) (also renamed exaptation by Gould and Vrba, 1982), arising as modifications, secondary uses, or side effects of preexisting traits. However, while the principle of tinkering may explain how consciousness appeared, we still need to ask how and why human consciousness became such a significant feature.

Another way to approach these questions is to consider whether consciousness carries a survival advantage that propelled its growth and influence beyond the original feature from which it evolved. These questions must be asked twice: first about the emergence of the ability to know, and second, equally important, about the capacity that accompanies knowing—namely, conscious control (and inhibition) of human perception and action. The discussion of these questions is beyond the scope of this essay.

Like any theory that aims to explain complex phenomena in the material world, the interface theory seeks to draw connecting lines between physical phenomena and partial insights related to consciousness, with the goal of creating a network with internal logic. This network of human consciousness is as coherent and complete as I could make it, but inevitably, it is limited in two major ways:

1. Even if many of the connections I propose between the various phenomena are correct, many aspects of these phenomena still require further clarification.
2. The interface theory addresses only a part, perhaps a small one, of the relevant phenomena necessary for a comprehensive understanding.

I will briefly mention three areas that were overlooked and require future study:

- The division of roles between hemispheres: The interface theory conceptualizes the brain vertically, from older to newer structures, without addressing the horizontal axis—the division between the right and left hemispheres. This division of roles has been studied mainly at a descriptive level (McGilchrist, 2009), but few, if any, attempts have been made to provide an evolutionary explanation for its emergence. The little we know about this division of roles suggests that it concerns both creativity and awareness, which justifies exploring its possible contribution to the characteristics of consciousness.
- The development of consciousness from birth to maturity: In addition to the vertical and horizontal axes, there is the axis of time. Studying the development of consciousness in the early years of life, before language becomes a sophisticated tool for expression, is challenging. Nevertheless, understanding how consciousness grows and develops within each individual could broaden and deepen our knowledge of its nature.
- The relationship between consciousness and the autonomic nervous system and non-neural control systems: Phenomena such as hypnosis and the placebo (and nocebo) effect challenge the relatively simple scheme I have presented here. The term autonomous implies that the autonomic nervous system is not controlled by consciousness, and indeed, this is usually the case. However, in a hypnotic state (and perhaps in other conditions), such control can be established instantaneously, without prior training. The placebo effect is another instance where conscious processes can influence autonomous and even non-neural systems, of which the person is usually unaware. These connections raise numerous questions that, for now, are beyond the explanatory power of any existing theory, including the interface theory.

## 4 Conclusion

The interface hypothesis of consciousness proposes a novel perspective on the evolution and function of human consciousness. Unlike traditional theories that view consciousness as an evolutionary upgrade of pre-existing cognitive skills, this hypothesis suggests that consciousness emerged as a byproduct of an advanced mammalian innovation-generating mechanism, based on the unpredictable, chaotic and exploratory functioning of the neocortex.

The hypothesis posits that the serial functioning of the interface evolved to crop and sort the chaotic outputs of the neocortex and communicate them to the control system, ensuring that novel solutions could be implemented in a stable and controlled way. According to this theory, human-type consciousness emerged as a side effect of this interface. Over time, following the extreme growth and strengthening of the innovation activity, this serially functioning system became powerful enough to expand its influence over other cognitive processes such as perception, motion, emotion, memory, and decision-making.

One of the principal traits that distinguishes humans from other animals is their extraordinary behavioral plasticity. Having to learn almost every motor, mental, and behavioral pattern, humans are at the same time open and vulnerable to external and internal agents that shape their behavior. Human consciousness is a powerful agent that has modified almost every human function. It has reshaped thinking and emotions, and it interferes with sensory-motor functions and even metabolic processes. All these functions, particularly the last two, can be carried out without consciousness being involved, but still, consciousness keeps interfering, even when there is no need for it.

The synergy between mammalian subcortical control systems and neocortical innovation generation, mediated by their interface, has evolved, been polished, and perfected over about 200 million years. The emergence of consciousness as a powerful independent agent that extends beyond the interface’s original role is a fairly recent event. This relatively short time, coupled with humans’ very slow reproduction rate, was insufficient for the perfection of consciousness’s interrelations with already established functions. Hence, its interaction with cognitive, motor, and even metabolic functions is quite often unhelpful or even detrimental.

Humans have extraordinary potential that may be realized in many different ways. However, a large part of this potential remains unfulfilled (James, 1907), and furthermore, many innate capacities are either inhibited or wrongly used. Apparently, this situation is not unchangeable. There are many instances in which alteration of conscious state (e.g., hypnosis and placebo) can modify and improve the functioning of almost every system, from internal secretion and immune systems, through motor and sensory functioning, to emotional and intellectual capacities. Such procedures are regarded as paramedical or “non-scientific” for the simple reasons that we do not know how they work, and because their effects are less specific and/or less measurable. However, since these phenomena cannot be ignored, science should find ways to embrace them.

Therefore, the challenge of understanding the nature of human consciousness can be more than just an intellectual pastime. A theory that can lead to a better understanding of the origin, setting, and mechanism of human consciousness may lead to practical results. Such understanding may refine already in-use techniques like biofeedback and mindfulness, find more accurate and efficient ways to achieve placebo-like effects, and may even replace dubious practices like faith-healing with more objective techniques that do not depend on the personalities or emotional states of the healer and patient. If it eventually turns out that the interface theory does indeed reflect reality, it may become part of humanity’s toolbox. It may contribute to the development of techniques that liberate and enhance inhibited human capacities of both body and mind.

## Data Availability

The original contributions presented in the study are included in the article/supplementary material, further inquiries can be directed to the corresponding author.
